# Neutrophil-to-Lymphocyte Ratio Is a Predictive Biomarker in Patients with Epidermal Growth Factor Receptor (EGFR) Mutated Advanced Non-Small Cell Lung Cancer (NSCLC) Treated with Tyrosine Kinase Inhibitor (TKI) Therapy

**DOI:** 10.3390/cancers13061426

**Published:** 2021-03-20

**Authors:** Nicole K. Yun, Sherin J. Rouhani, Christine M. Bestvina, Ethan M. Ritz, Brendan A. Gilmore, Imad Tarhoni, Jeffrey A. Borgia, Marta Batus, Philip D. Bonomi, Mary Jo Fidler

**Affiliations:** 1 Department of Internal Medicine, Rush University Medical Center, Chicago, IL 60612, USA; nicole_k_yun@rush.edu; 2 Section of Hematology/Oncology, Department of Medicine, The University of Chicago Comprehensive Cancer Center, Chicago, IL 60637, USA; Sherin.rouhani@uchospitals.edu (S.J.R.); cbestvina@medicine.bsd.uchicago.edu (C.M.B.); 3 Bioinformatics and Biostatistics Core, Rush University Medical Center, Chicago, IL 60612, USA; Ethan_M_Ritz@rush.edu; 4 Hematology, Oncology and Cell Therapy, Department of Medicine, Rush University Medical Center, Chicago, IL 60612, USA; Brendan_gilmore@rush.edu (B.A.G.); Marta_Batus@rush.edu (M.B.); Philip_Bonomi@Rush.edu (P.D.B.); 5 Cell & Molecular Medicine, Pathology, Rush University Medical Center, Chicago, IL 60612, USA; Imad_Tarhoni@rush.edu (I.T.); Jeffrey_A_Borgia@rush.edu (J.A.B.)

**Keywords:** tyrosine kinase inhibitor (TKI), neutrophil-to-lymphocyte ratio (NLR), predictive biomarker, body mass index (BMI), epidermal growth factor receptor (EGFR), advanced non-small cell lung cancer (NSCLC)

## Abstract

**Simple Summary:**

Though patients with sensitizing epidermal growth factor receptor (*EGFR*) mutated non-small cell lung cancer have been known to have rapid and durable responses to tyrosine kinase inhibitors, a subset of patients have inferior outcomes and may benefit from therapy escalation. Neutrophil-to-lymphocyte ratio (NLR) has been recognized as one indicator of poor prognosis in patients with various cancers, including non-small cell lung cancer. It is associated with cancer cachexia and treatment resistance. The aim of our study is to assess the serial predictive value of NLR and other markers of cachexia and chronic inflammation in the subset of patients with advanced non-small cell lung cancer who harbor the epidermal growth factor receptor mutation and who are undergoing treatment with tyrosine kinase inhibitor therapy.

**Abstract:**

Background: First-line treatment for patients with non-small cell lung cancer (NSCLC) with a sensitizing epidermal growth factor receptor (*EGFR*) mutation is a tyrosine kinase inhibitor (TKI). Despite higher response rates and prolonged progression free survival (PFS) compared with platinum doublet chemotherapy, a subset of these patients do not receive prolonged benefit from these agents. We investigate if the neutrophil-to-lymphocyte ratio (NLR) and other markers of cachexia and chronic inflammation correlate with worse outcomes in these patients. Methods: This study is a retrospective review of 137 patients with advanced *EGFR*-mutated NSCLC treated with TKIs at Rush University Medical Center and University of Chicago Medicine from August 2011 to July 2019, with outcomes followed through July 2020. The predictive value of NLR and body mass index (BMI) was assessed at the start of therapy, and after 6 and 12 weeks of treatment by univariable and multivariable analyses. Results: On univariable analysis, NLR ≥ 5 or higher NLR on a continuous scale were both associated with significantly worse PFS and overall survival (OS) at treatment initiation, and after 6 or 12 weeks of treatment. On multivariable analysis, NLR ≥ 5 was associated with increased risk of death at 12 weeks of therapy (HR 3.002, 95% CI 1.282–7.029, *p* = 0.011), as was higher NLR on a continuous scale (HR 1.231, 95% CI 1.063–1.425, *p* = 0.0054). There was no difference in PFS and OS and amongst BMI categories though number of disease sites and Eastern Cooperative Oncology Group (ECOG) performance status was associated with worse PFS and OS. Conclusions: Patients with NLR ≥ 5 have a worse median PFS and median OS than patients with NLR < 5. NLR may have value as a predictive biomarker and may be useful for selecting patients for therapy intensification in the front-line setting either at diagnosis or after 12 weeks on therapy. NLR needs to be validated prospectively.

## 1. Introduction

Lung cancer remains the leading cause of cancer death among both men and women worldwide [1]. Lung cancer patients with sensitizing driver mutations in the epidermal growth factor receptor (*EGFR)* gene make up approximately 15% of the NSCLC patient overall. In recent years, the prognosis and quality of life of patients with advanced NSCLC harboring *EGFR* mutations has changed dramatically due to the incorporation of tyrosine kinase inhibitors (TKIs), now standard of care as first-line therapy [2]. Median overall survival (mOS) has increased to 38.6 months with osimertinib with the additional benefit of a more tolerable side effect profile compared with prior generation TKI therapy or cytotoxic chemotherapy [3]. While there is a group of patients with exon 20 insertions and other uncommon *EGFR* mutations that are not sensitive to the current generations of TKIs, there is also a group of patients with known sensitizing mutations who, nonetheless, exhibit primary resistance and can progress in as little as 3 months [4].

Cancer-associated inflammation in the tumor microenvironment and systemic inflammation can lead to tumor growth and therapy resistance. Myeloid-derived suppressor cells (MDSCs) and tumor associated neutrophils (TAN) can inhibit the immune response through a variety of direct and soluble mechanisms. Lymphocytes consist of B cells which produce antibodies, and T cells which help activate B cells and which also have direct cytotoxic activity against tumor cells. Therefore, the immune response profile can serve as a useful tool to predict patient prognosis and response to treatment. The neutrophil-to-lymphocyte ratio (NLR) and platelet-to-lymphocyte ratios (PLR) have both been investigated as potential prognostic and predictive biomarkers that are convenient and easy to obtain with routine lab tests. An elevated NLR has emerged as an indicator of poor prognosis across a variety of tumor types [5,6,7,8]. Wang et al conducted a 2019 meta-analysis looking at pretreatment NLR for NSCLC patients receiving systemic therapy including chemotherapy, immunotherapy and targeted therapy. The authors reported that an elevated pretreatment NLR is consistently associated with inferior survival [9].

More recently, reports have begun to comment on NLR as a biomarker for predicting prognosis and response to treatment in patients with advanced, *EGFR*-mutated NSCLC who are treated with TKI therapy [2,8,10,11]. Most of the studies investigating NLR in *EGFR*-mutated NSCLC have been conducted in predominantly Asian populations [8,10,11,12,13]. Although there have also been similar reports in European [14] and Australian populations [15], there is little information in an African American population. Prior studies looking at patients with *EGFR*-mutated NSCLC undergoing treatment with TKIs have used variable NLR cutoff values, frequently chosen to reflect the top quartile of the study population or to maximize the receiver operating characteristic (ROC) curve. These cutoffs generally range from 3–5, but an optimal value has not been validated. In concordance with several prior studies [5,6,7,8,9,10,11,12,13,14,15], we have elected to use an NLR cutoff of 5 to provide greater discriminatory potential. Additionally, there have been few studies to date including patients treated with osimertinib, a third generation TKI which was FDA approved in 2017. The purpose of our study is to validate the NLR as a predictive indicator at different time points during therapy, and to examine other factors potentially contributing to heightened systemic inflammatory response such as baseline number of metastatic sites and therapy-related toxicities. Since cancer cachexia can be an indicator of a pro-inflammatory state and BMI < 18.5 has also been reported as a poor prognostic marker [10], we also investigate the relationship between changes in BMI with changes in NLR, progression free survival (PFS), and overall survival (OS).

## 2. Materials and Methods

### 2.1. Patient Selection and Data Collection

We retrospectively analyzed 137 patients with advanced non-small cell lung cancer treated at Rush University Medical Center or University of Chicago Medicine from August 2011 to July 2019. Outcomes were followed through 1 July 2020. Patients aged 18 years or older with advanced non-small cell lung cancer and a targetable *EGFR* mutation, including exon 19 deletions, L858R, L861Q, S768I, G709A, and G719S mutations, were included. *EGFR* mutations with known TKI resistance such as exon 20 insertions were excluded. All patients received oral EGFR tyrosine kinase inhibitor therapy (erlotinib, gefitinib, afatinib, osimertinib), and progression free survival and overall survival were measured from time of first TKI treatment.

For each patient, the NLR was recorded at the time of TKI initiation, 6 weeks after starting TKI and 12 weeks after starting TKI. The values were taken from routine complete blood count (CBC) with differential drawn closest to the respective time point ± 30 days. Most CBC were drawn at outpatient appointments. Treatment responses were evaluated using computed tomography (CT) every 8–12 weeks per routine clinical care. For those who experienced progression while on TKI, we recorded the NLR at the time of progression and at 6 weeks prior to progression. Body mass index (BMI) of the patient was also collected at the time of diagnosis, time of therapy initiation, 6 weeks after therapy, 12 weeks after therapy, and at progression and 6 weeks prior, if applicable.

We defined PFS as time from start of TKI to time of disease progression on imaging, such as interval increase in size of primary or metastatic lesion, increase in size of malignant pleural effusion, or new metastatic disease that resulted in a clinical change in therapy. Due to the number of patients having nonmeasurable disease and nontarget lesions, we were unable to use Response Evaluation Criteria in Solid Tumors (RECIST) to define tumor progression. In cases of oligoprogression treated with local ablative therapy, disease was considered progressive only if the scan results triggered a change in systemic therapy, including switching to a third generation TKI or chemotherapy. OS was calculated as time from start of TKI until time of death or last clinical follow-up.

Drug toxicities attributed to TKI therapy were graded by severity in accordance with version 5 of the Common Terminology Criteria for Adverse Events (CTCAE). We recorded the highest grade experienced by the patient throughout the entire duration of treatment with TKI.

Baseline number of disease sites was used to quantify disease burden and account for varying degrees of tumor involvement between patients. By referring to initial staging CT, positron emission tomography (PET) scan and/or magnetic resonance imaging (MRI) at time of diagnosis, the following sites of disease were recorded: lung, regional lymph nodes (LN), distant LN, pleura, heart/pericardium, bone, liver, adrenal, brain, and other. The distinction between regional and distant LN were made based on the TNM staging system.

### 2.2. Statistics

PFS and OS were computed using the Kaplan-Meier method and compared using the log rank test. Spearman rho (*ρ*) was used to correlate number of disease sites with NLR at diagnosis. T-tests were used to compare mean NLR between patients with any high grade (≥3) toxicity versus those with no or low (grades 0–2) toxicity. To assess whether or not a relationship existed between NLR and BMI throughout the course of TKI therapy, changes in NLR and BMI were calculated by subtracting the value at treatment start from the values at 6 or 12 weeks, and the relationship between the change in NLR vs. BMI was analyzed using Pearson correlation. Cox regression was used to assess correlation between change in NLR and BMI with PFS and OS at 6 weeks and 12 weeks. Univariable and multivariable analysis was also used to compare PFS and OS in patients with varying baseline characteristics: age at diagnosis, gender, race, smoking status, BMI, total number of metastatic sites, central nervous system (CNS) metastases, first-line TKI, other systemic treatment prior to TKI initiation, Eastern Cooperative Oncology Group (ECOG) performance status, toxicity grade, and mutation. Multivariable cox regression analysis was performed to assess the independent predictive value of NLR as well as significance of other baseline characteristics. All analyses were conducted using R version 4.0.3.

## 3. Results

### 3.1. Patient Characteristics

In total, 137 patients treated at Rush University Medical Center or University of Chicago Medicine from August 2011 to July 2019 were retrospectively analyzed. Baseline demographics with classification criteria are shown in Table 1. Median age at diagnosis was 63.9 years, with an interquartile range of 56.7 to 71.9 years. Our patient population was predominantly female (102/137 patients, 74.4%), and 45.2% were never smokers. Patient race was 62.8% white, 21.9% black or African American, and 8.8% Asian. A total of 51.1% of patients had an exon 19 deletion, 33.3% had L858R, and 5.0% had T790M on their initial genetic testing.

The majority (54.7%) of patients received first-line treatment with erlotinib (Table 1). Importantly, 26% of patients received first-line treatment with osimertinib. The proportion of patients on each therapy with an NLR < 5 compared to NLR ≥ 5 was relatively similar at the time of therapy initiation (Table 2).

### 3.2. Univariable Analysis on NLR and Survival Outcomes

**Figure 1:**
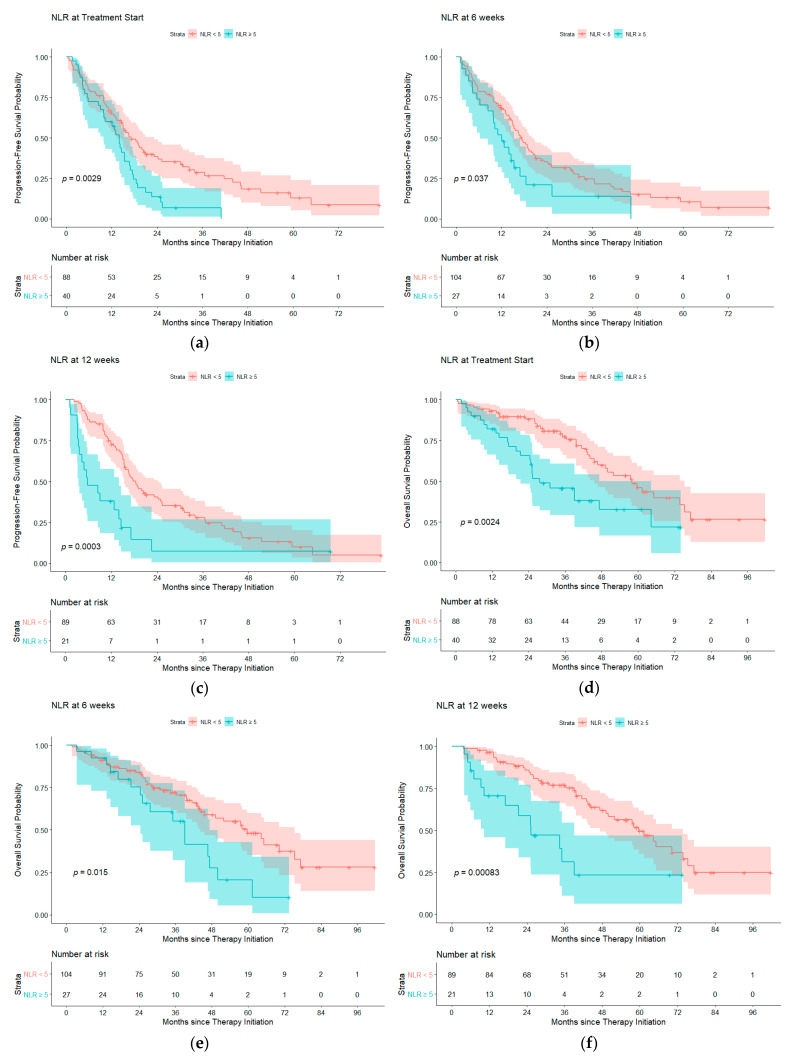
Progression free survival probability for NLR < 5 (blue) compared to NLR ≥ 5 (red) at (**a**) treatment start, (**b**) 6 weeks of therapy and (**c**) 12 weeks of therapy. Overall survival probability for NLR < 5 compared to NLR ≥ 5 at (**d**) treatment start, (**e**) 6 weeks of therapy and (**f**) 12 weeks of therapy. Abbreviations: NLR, neutrophil-to-lymphocyte ratio.

Median follow-up time from date of TKI initiation was 36.6 months, with a maximum of 103.4 months and a minimum of 6.3 months. The PFS and OS curves are depicted in Figure 1 with results summarized in Table 3. At therapy start, patients with NLR < 5 (*n* = 88) had a median progression free survival (mPFS) of 17.2 months and a mOS of 58.0 months, while those with NLR ≥ 5 (*n* = 40) had a mPFS of 14.0 months (*p* = 0.0029) and mOS of 27.6 months (*p* = 0.0024). After 6 weeks of treatment, patients with NLR < 5 (*n* = 104) had a mPFS of 17.4 months and mOS of 59.7 months, while those with NLR ≥ 5 (*n* = 27) had a mPFS of 12.1 months (*p* = 0.0037) and a mOS of 39.1 months (*p* = 0.015). After 12 weeks of treatment, patients with NLR < 5 (*n* = 89) had a mPFS of 18.4 months and mOS of 59.7 months, while those with NLR ≥ 5 (*n* = 21) had a mPFS of 5.8 months (*p*= 0.0003) and a mOS of 25.2 months (*p* = 0.00083). At time of progression, patients with NLR < 5 (*n* = 71) had a mOS of 33.4 months, while those with NLR ≥ 5 had a mOS of 10.2 months (*p* = 0.00022). Survival outcomes were also analyzed using NLR ≥ 3 and NLR < 3 to assess its predictive value at another commonly used threshold. A similar association was seen where NLR ≥ 3 predicted worse OS at treatment initiation and after 12 weeks; results are summarized in Figure S1 and Table S1.

When assessing NLR as a continuous variable without adjusting for covariates, there was an increased risk for progression at treatment start (hazard ratio (HR) 1.050, 95% confidence interval (CI) 1.010–1.092, *p* = 0.0137), at 6 weeks (HR 1.073, 95% CI 1.022–1.127, *p* = 0.0047) and at 12 weeks (HR 1.169, 95% CI 1.080–1.266, *p* = 0.0001). There was also an increased risk for death at treatment start (HR 1.060, 95% CI 1.013–1.109, *p* = 0.0113), 6 weeks (HR 1.087, 95% CI 1.027–1.152, *p* = 0.0043) and at 12 weeks (HR 1.181, 95% CI 1.063–1.312, *p* = 0.002)

Patients who had a decrease in NLR did not have significantly better PFS or OS compared to patients whose NLR increased at either 6 weeks (*p* = 0.29) or 12 weeks (*p* = 0.52). Furthermore, there was no significant change in the NLR from the start of treatment to 6 weeks prior to progression (mean = −0.9702, standard deviation (SD) = 5.6207, *n* = 86, *p* = 0.1786), or to the time of progression (mean = 0.4406, SD = 7.3476, *n* = 96, *p* = 0.5582).

### 3.3. Univariable Analysis on BMI and Studied Outcomes

There was no significant difference between patients classified as underweight (BMI ≤ 18.5), normal weight (BMI >18.5 to ≤ 25), overweight (BMI > 25 to ≤ 30) and obese (BMI > 30), in PFS and OS at therapy start, 6 weeks and 12 weeks. There was no statistically significant difference in OS between BMI categories at time of progression (Figure S2, Table S2). The change in BMI after 6 weeks from therapy start and 12 weeks from therapy start was not associated with PFS (*p* = 0.331 for 6 weeks, *p* = 0.439 for 12 weeks) or OS (*p* = 0.107 for 6 weeks, *p* = 0.174 for 12 weeks). However, there was an association between BMI change at progression and OS after progression (HR = 0.8371, *p* = 0.00336) (Table S3).

No correlation was found between baseline BMI and NLR (*r* = −0.145, *p* = 0.104). Changes in BMI did not correlate with changes in NLR at 6 weeks of therapy (*r* = −0.13, *p* = 0.16) but significantly correlated at 12 weeks (*r* = −0.21, *p* = 0.03) (Figure S3).

### 3.4. Total Number of Disease Sites, Adverse Drug Events and ECOG Score on NLR

There was a modest correlation between baseline number of disease sites and NLR at therapy start (Spearman’s *ρ* = 0.272, *p* = 0.002). Throughout the course of treatment, 72% of patients reported either no or low-grade adverse drug events (grade 0–2), while 28% of patients experienced high grade (≥3) adverse events. There was no difference in NLR at 6 weeks or 12 weeks between patients with high grade versus no or low-grade drug toxicities at 6 weeks (*p* = 0.28) or 12 weeks (*p* = 0.30), and development of grade 3–5 toxicities did not affect PFS (*p* = 0.09) or OS (*p* = 0.8). There was no correlation between ECOG score and NLR at therapy start (*r* = 0.095, *p* = 0.289)

### 3.5. Multivariable Analysis on Baseline Patient Characteristics and Survival Outcomes at Treatment Start

Multivariable cox regression looking at baseline patient characteristics and their relation to OS and PFS are summarized in Table 4. NLR ≥ 5 compared to NLR < 5 at the time of therapy initiation was not associated with increased risk for death or progression after controlling for baseline patient characteristics. Age at diagnosis and gender were not associated with increased risk for progression or for death. African American race did not portend worse OS or PFS when compared to white; however, Asian and other race was associated with worse OS. Baseline number of disease sites at therapy start was an independent factor associated with decreased OS (HR 1.825, 95% CI 1.328–2.507, *p* = 0.0002) and decreased PFS (HR 1.473, 95% CI 1.172–1.851, *p* = 0.0009). ECOG score of 2–3 compared to 1–2 was strongly associated with decreased OS (HR 4.390, 95% CI 2.037–9.458, *p* = 0.0002) and decreased PFS (HR 3.435, 95% CI 1.779–6.634, *p* = 0.0002). Patients harboring an exon 19 mutation had lower risk for death than patients without the mutation (HR 0.213, 95% CI 0.048–0.948, *p* = 0.042), and patients with G719X mutation had an increased risk for death compared to those without (HR 6.239, 95% CI 1.026–37.931, *p* = 0.0047). Greater than 15 pack year smoking history was associated with worse OS (HR 4.574, 95% CI 1.835–11.401, *p* = 0.001) but not PFS when compared to nonsmokers.

### 3.6. Multivariable Analysis of NLR at Treatment Start, 6 Weeks and 12 Weeks of Therapy

Multivariable analysis of NLR ≥ 5 compared to <5 did not show increased risk for progression at treatment start (HR 1.077, 95% CI 0.558–1.456 *p* = 0.801), 6 weeks (HR 1.032, 95% CI 0.560–1.902, *p* = 0.921) or 12 weeks of therapy (HR 2.075, 95% CI 0.976–4.412, *p* = 0.058). When looking at OS, NLR ≥ 5 compared to <5 did not show an increased risk for death at therapy start (HR 1.935, 95% CI 0.911–4.108, *p* = 0.086) or 6 weeks (HR 1.419, 95% CI 0.645–3.124, *p* = 0.384); however, NLR ≥ 5 was associated with increased risk of death at 12 weeks of therapy (HR 3.002, 95% CI 1.282–7.029, *p* = 0.011).

Multivariable analysis of NLR using cutoff value of ≥3 compared to <3 did not show increased risk of progression at treatment start (HR 1.041, 95% CI 0.622–1.742, *p* = 0.879), 6 weeks of therapy (HR 0.911, 95% CI 0.567–1.464, *p* = 0.700) or 12 weeks (HR 0.849, 95% CI 0.489–1.475, *p* = 0.562) nor increased risk of death at 6 weeks (HR 1.683, 95% CI 0.833–3.398, *p* = 0.147). NLR ≥ 3 was significantly associated with reduced OS at treatment start (HR 2.374, 95% CI 1.161–4.852, *p* = 0.018) and at 12 weeks of therapy (HR 2.337, 95% CI 1.126–4.849, *p* = 0.023)

Multivariable analysis assessing NLR as a continuous variable did not show increased risk of progression at treatment start (HR 0.995, 95% CI 0.934–1.060, *p* = 0.871), 6 weeks (HR 1.011, 95% CI 0.946–1.080, *p* = 0.746) or 12 weeks (HR 1.123, 95% CI 0.975–1.293, *p* = 0.108). When assessing NLR as a continuous variable on OS, there was no statistically significant increased risk of death at treatment start (HR 1.007, 95% CI 0.943–1.074, *p* = 0.839) or at 6 weeks (HR 1.043, 95% CI 0.970–1.121, *p* = 0.255); however, NLR was found to be significant for OS at the 12-week mark (HR 1.231, 95% CI 1.063–1.425, *p* = 0.0054)

## 4. Discussion

The NLR provides an easily measurable biomarker that warrants further investigation as a predictive biomarker. The biologic reasons why the NLR may be predictive are likely multifactorial. One possibility is that a higher NLR could reflect a more aggressive underlying tumor phenotype. Tumors secrete G-CSF and other soluble mediators that stimulate hematopoiesis [16], and if more aggressive tumors secrete cytokines predominantly skewing towards increased neutrophil production this could result in an elevated NLR [17]. Additionally, there may be a causal relationship where increased numbers of neutrophils compared to lymphocytes further inhibits the anti-tumor response. Activated cytotoxic CD8^+^ T cells directly kill tumor cells. The activity of these T cells can be suppressed either by regulatory T cells or myeloid-derived suppressors cells (MDSC). MDSCs are a heterogenous group of cells that can be derived from either a monocytic or neutrophilic lineage [18]. Neutrophils can secrete soluble factors including reactive oxygen species (ROS) and arginase 1 to inhibit T cells [19]. Tumor associated neutrophils (TAN) are another population of cells that can be either pro- or anti-tumorigenic. Neutrophil extracellular traps (NETs) have been associated with increasing the metastatic potential of tumor cells and neutrophils can also secrete factors such as VEGF which promote tumor angiogenesis [20,21,22]. Future work is needed to further understand the biologic underpinnings of the clinical correlation between NLR and prognosis and to identify novel strategies to target the EGFR tumor microenvironment in patients with high NLR.

One question is whether a change in the NLR during treatment is related to upcoming progression. There was no significant change in the NLR from the start of treatment to 6 weeks prior to progression (mean = −0.9702, SD = 5.6207, *n* = 86, *p* = 0.1786), or to the time of progression (mean = 0.4406, SD = 7.3476, *n* = 96, *p* = 0.5582). When comparing NLR at progression and NLR at start of therapy amongst patients who progressed (*n* = 96), NLR on average increased; however, this finding was not statistically significant (mean 0.441, SD 7.348, *p* = 0.558). There was also no significant difference in PFS and OS for patients whose NLR decreased at 6 and 12 weeks compared to those whose NLR increased at 6 and 12 weeks, which led us to conclude that changes in NLR do not have any utility in predicting progression or prognosis.

Another factor that has been shown to affect prognosis in lung cancer is BMI. No significant difference in OS was noted between the different BMI groups at therapy start, 6 weeks, 12 weeks and progression. After adjusting for covariates, BMI was not an independent predictive factor for OS and PFS when comparing patients classified as underweight, overweight and obese to patients with normal BMI. A change in BMI after 6 weeks or 12 weeks from therapy start was not correlated with PFS (*p* = 0.331 for 6 weeks, *p* = 0.439 for 12 weeks) or OS (*p* = 0.107 for 6 weeks, *p* = 0.174 for 12 weeks); however, there was a correlation noted between BMI change (from therapy start to progression) and OS after progression (HR = 0.8371, *p* = 0.00336.) One potential explanation for these findings is that cancer-mediated cachexia is a slow process that becomes evident over longer periods of time as opposed to within the first few months of initiating therapy.

Prognostic stratification of patients by NLR has therapeutic implications. Combining TKI therapy with chemotherapy in patients with an extensive disease burden may offer improvements in PFS and OS [23]. FLAURA2 is a phase III randomized trial underway to determine if combining osimertinib with carboplatin/pemetrexed improves PFS and OS (ClinicalTrials.gov accessed on 22 March 2021 Identifier NCT04035486). Antiangiogenic agents are also being pursued (ClinicalTrials.gov accessed on 22 March 2021 Identifier NCT04181060) with a recent publication suggesting bevacizumab and osimertinib led to a favorable 1-year PFS [24] and other trials that are ongoing as well are trials adding the anti-angiogenic agentand ramucirumab (ClinicalTrials.gov accessed on 22 March 2021 Identifier NCT03909334). Studying the NLR in patients treated with angiogenic agents would also be useful in light of recent FDA approval of ramucirumab with erlotinib in front-line treatment of metastatic NSCLC with *EGFR* exon 19 deletions or exon 21 (L858R) mutations [25]. Using the NLR at 12 weeks of therapy to identify patients with a poor prognosis may help select the population most likely to benefit from early intensification of treatment. Additionally, as novel therapeutics targeting the suppressive effects of neutrophils are developed, the NLR could be used as a predictive factor to stratify patients who may be most likely to benefit from these strategies in combination with a TKI.

Our study does have several limitations. It is a retrospective study, and information was obtained from standard clinical documentation. Additionally, we used clinical notes and change in therapy to determine progression instead of RECIST criteria however as many patients are kept on TKIs with minimal progression and with the application of local therapies, we feel this is a minor weakness. Some patients did not have the NLR measurements available at all timepoints examined or had NLR examined from a hospital stay. We feel this is a minor weakness as we did not see a correlation between NLR and ECOG performance status, which could reflect underlying reasons for hospital admission We also were not able to analyze effects of co-mutations such as those in *Rb* and p53 or circulating tumor DNA clearance which have been shown to portend poor prognosis [26,27]. Furthermore, since toxicity data was taken from the treating physician’s medical record notes it may not be as complete as toxicity data which is prospectively documented for patients enrolled in a clinical trial. Finally, during the time course of our study the standard of care TKI shifted to the third-generation drug, osimertinib. While over 25% of our cohort was treated with osimertinib as their initial TKI, our study also included patients treated with older generation TKIs and some who had initiated treatment with an earlier generation TKI while awaiting approval for a newer generation.

Previous studies examining the effect of NLR on an *EGFR*-mutated population have largely been done in Asian populations [13]. One strength of our study is that data is pooled from two institutions in Chicago with a racially diverse patient population, and the patient race distribution was reflective of the study location. Our study cohort was 22% African American, which is important given the paucity of data that exists which addresses racial disparities among patients with *EGFR* mutated lung cancer. Cheng et al (2020) reported inferior survival among black patients with NSCLC with *EGFR* mutations as compared to non-black patients, with 2-year survival rates being 33% versus 61%, respectively (*p* = 0.001) [28]. Shorter survival was also observed for those patients treated with targeted EGFR therapy. Earlier studies have hypothesized that the survival discrepancy may be more a consequence of socioeconomic disparities as opposed to genetic factors [29,30,31]. Most of these studies were obtained before targeted therapy became standard of care. We did not find a statistically significant difference in PFS and OS between African American and non-African American patients but, nevertheless, further investigation on racial disparities and survival outcomes in this new TKI era are warranted.

Prior studies have looked solely at pretreatment NLR and have found that pretreatment NLR is an independent predictor of PFS and OS [9,32]. We did not observe NLR at the pretreatment stage (i.e., time of therapy initiation) to be an independent predictive factor as prior studies have reported, possibly due to our limited sample size and the strong association between baseline number of disease sites with OS and PFS, and ECOG score with OS and PFS. Our work nevertheless highlights the value of serial NLR measurements collected at multiple time points throughout TKI therapy and not just at the pre-treatment stage, as NLR is a dynamic value that is expected to fluctuate throughout disease course and treatment. We validate previous studies demonstrating that elevated NLR is a poor prognostic sign and further extend this finding to include patients treated with the third generation TKI osimertinib.

## 5. Conclusions

The results of our study supporting NLR as a predictive biomarker for patients with advanced stage *EGFR* mutated NSCLC on TKI therapy is concordant with what has been previously described in the literature. We highlight the predictive value of NLR on OS particularly at the 12-week mark after initiation of therapy. Based on these results, increased frequency of monitoring and potential therapeutic escalation may be beneficial in patients with elevated NLR ≥ 5, particularly after receiving 12 weeks of TKI therapy.

## Figures and Tables

**Table 1:** Baseline patient characteristics and distribution divided by neutrophil-to-lymphocyte ratio (NLR) at therapy start.

Patient Characteristics	NLR ^1^ < 5	NLR ≥ 5	NLR N/A ^2^	Total
Age at Diagnosis				
<65	46	23	4	73
≥65	42	17	5	64
Gender				
Male	19	14	2	35
Female	69	26	7	102
Race				
White	53	26	7	86
Asian	9	3	0	12
Black/African American	20	8	2	30
Other	4	1	0	5
More than one	1	0	0	1
Not reported	1	2	0	3
Smoking Status				
Never	41	17	4	62
≤15 pack years	30	12	2	44
>15 pack years	17	10	3	30
Former (not counted)	0	1	0	1
Baseline BMI ^3^				
≤18.5	0	2	0	2
>18.5 to ≤25	36	23	3	62
>25 to ≤30	31	10	3	44
>30	20	5	3	28
Not available	1	0	0	1
Number of Metastatic Sites				
1	8	1	1	10
2	29	1	3	33
3	25	14	3	42
4	16	11	1	28
5	8	8	0	16
6	0	1	0	1
7	2	4	1	7
CNS ^4^ Metastases				
Present	25	21	1	47
Not present	63	19	8	90
Histology				
Adenocarcinoma	86	40	9	135
Mixed/poorly differentiated NSCLC ^5^	2	0	0	2
Baseline Mutations				
Exon 19 Deletion (Total with All Co-mutations)	49	21	2	72
Exon 19 alone	46	21	2	69
+T790M	2	0	0	2
+*EGFR* ^6^ amplification	1	0	0	1
+L858R	1	0	0	1
L858R (Total with All Co-mutations)	30	15	2	47
L858R alone	24	14	2	40
+T790M	4	1	0	5
+Exon 19	1	0	0	1
+L833V	1	0	0	1
T790M (Total with All Co-mutations)	6	1	0	7
+Exon 19	2	0	0	2
+L858R	4	1	0	5
G719X (Total with All Co-mutations)	6	3	0	9
G719A alone	1	1	0	2
G719A + L833_V834delinsFL	1	0	0	1
G719A + S768I	1	0	0	1
G719C + S768I	1	0	0	1
G719A + E709A	1	0	0	1
G719S + G709A	1	0	0	1
G719S + E709A	0	2	0	2
S768I (Total with All Co-mutations)	3	0	1	4
S768I alone	1	0	1	2
S768I +G719X	2	0	0	2
L861Q Alone	1	0	1	2
Others				
Exon 18 del *p*.E709_T710delinsD	0	0	1	1
LUL: Exon 19; RUL: pT790M and L861Q	0	0	1	1
*EGFR* amplification and E709_T710 > D;	0	0	1	1
Not known	1	1	0	2
First-line TKI ^7^				
Erlotinib	48	21	7	76
Osimertinib	26	9	1	36
Afatinib	13	10	1	24
Gefitinib	1	0	0	1
TKI Began 1+ Years after Advanced Stage Diagnosis				
Yes	7	3	0	10
No	81	37	9	127
Other Systemic Treatment for Metastatic Disease before Starting TKI				
Yes	28	8	0	36
No	60	32	9	101
ECOG ^8^ Score				
0	30	9	5	44
1	49	23	4	76
2	6	6	0	12
3	2	2	0	4
Not reported	1	0	0	1
Toxicity Grade				
Grade 0–2	62	31	5	98
Grade 3–4	25	8	4	37
Grade 5	1	1	0	2

1. NLR, neutrophil-to-lymphocyte ratio; 2. BMI, body mass index; 3. N/A, not available; 4. CNS, central nervous system; 5. NSCLC, non-small cell lung cancer; 6. EGFR, epidermal growth factor receptor; 7. TKI, tyrosine kinase inhibitor; 8. ECOG, Eastern Cooperative Oncology Group.

**Table 2:** Distribution of TKI use at time of therapy start, 6 weeks and 12 weeks for patients with NLR < 5 and NLR ≥ 5.

TKI	Therapy Start		6 Weeks		12 Weeks	
Drug	NLR < 5	NLR ≥ 5	NLR < 5	NLR ≥ 5	NLR < 5	NLR ≥ 5
Afatinib	15%	15%	19%	12%	19%	15%
Erlotinib	54.50%	32%	50%	69%	59%	50%
Gefitinib	1%	0%	1%	0%	1%	0%
Osimertinib	29.50%	53%	30%	19%	21%	35%

**Table 3:** Progression free survival (PFS) and overall survival (OS) for patients with NLR ≥ 5 and < 5 at therapy start, 6 weeks and 12 weeks after beginning treatment.

Outcome	NLR	Measurement Time	*n*	Median (Months)	95% CI	*p*-Value
OS	<5	Treatment Start	88	58	(45.5, 75.3)	0.0024
OS	≥5	Treatment Start	40	27.6	(21.3, 47.3)	
OS	<5	6 weeks after Treatment Start	104	59.7	(44.2, 75.3)	0.015
OS	≥5	6 weeks after Treatment Start	27	39.1	(24.3, 47.3)	
OS	<5	12 weeks after Treatment Start	89	59.7	(47.3, 73.8)	0.00083
OS	≥5	12 weeks after Treatment Start	21	25.2	(10.2, 39.1)	
PFS	<5	Treatment Start	88	17.2	(13.3, 23.3)	0.0029
PFS	≥5	Treatment Start	40	14	(9.8, 16.7)	
PFS	<5	6 weeks after Treatment Start	104	17.4	(14.9, 20.5)	0.037
PFS	≥5	6 weeks after Treatment Start	27	12.1	(6.3, 15.4)	
PFS	<5	12 weeks after Treatment Start	89	18.4	(15.4, 24.3)	0.0003
PFS	≥5	12 weeks after Treatment Start	21	5.8	(3.4, 14.0)	
OS	<5	Progression	71	33.4	(25.2, 42.5)	0.00022
OS	≥5	Progression	31	10.2	(3.7, 17.9)	

Abbreviations: CI, confidence interval; PFS, progression free survival; OS, overall survival.

**Table 4:** Multivariable Cox regression analysis comparing impact of baseline characteristics on overall survival (OS) and progression free survival (PFS) at treatment start.

OS				PFS			
Group	HR ^1^	95% CI	*p*	Group	HR	95% CI	*p*
NLR (pre-treatment)				NLR (pre-treatment)			
≥5 (vs. <5)	1.935	(0.911, 4.106)	0.086	≥5 (vs. <5)	1.077	(0.602, 1.925)	0.803
Age				Age			
≥65 (vs. <65)	1.107	(0.574, 2.135)	0.7615	≥65 (vs. <65)	0.902	(0.558, 1.456)	0.672
Gender				Gender			
Male (vs. Female)	0.892	(0.410, 1.942)	0.773	Male (vs. Female)	16.7	(14.5, 20.0)	0.052
Race				Race			
Asian (vs. White)	4.274	(1.399, 13.064)	0.011	Asian (vs. White)	0.951	(0.418, 2.167)	0.906
Black (vs. White)	0.998	(0.424, 2.305)	0.978	Black (vs. White)	0.72	(0.384, 1.348)	0.304
Other (vs. White)	5.169	(1.191, 22.428)	0.028	Other (vs. White)	1.413	(0.492, 4.061)	0.521
Smoking History				Smoking History			
≤15 pack years (vs. never)	2.036	(0.884, 4.688)	0.095	≤15 pack years (vs. never)	1.452	(0.801, 2.632)	0.219
>15 pack years (vs. never)	4.574	(1.835, 11.401)	0.001	>15 pack years (vs. never)	1.754	(0.816, 3.771)	0.15
BMI Therapy Start				BMI Therapy Start			
<18.5 (vs. 18.5 to ≤25)	3.85	(0.575, 25.762)	0.165	<18.5 (vs. 18.5 to ≤25)	1.601	(0.263, 9.751)	0.61
>25 to ≤30 (vs. 18.5 to ≤25)	1.205	(0.563, 2.577)	0.631	>25 to ≤30 (vs. 18.5 to ≤25)	1.314	(0.766, 2.253)	0.321
>30 (vs. 18.5 to ≤25)	0.487	(0.177, 1.337)	1.625	>30 (vs. 18.5 to ≤25)	1.155	(0.597, 2.234)	0.669
Number of Disease Sites (continuous)	1.825	(1.328, 2.507)	0.0002	Number of Disease Sites (continuous)	1.473	(1.172,1.851)	0.0009
TKI				TKI			
Afatinib (vs. osimertinib)	1.123	(0.310, 4.006)	0.86	Afatinib (vs. osimertinib)	2.267	(0.958, 5.363)	0.063
Erlotinib (vs. osimertinib)	1.562	(0.586, 4.162)	0.372	Erlotinib (vs. osimertinib)	2.925	(1.501, 5.699)	0.0016
TKI Began 1+ Years after Stage IV Diagnosis	0.529	(0.126, 2.224)	0.385	TKI Began 1+ Years after Stage IV Diagnosis	0.643	(0.238, 1.739)	0.385
Received Other Systemic Treatment before TKI	2.253	(0.976, 5.203)	0.058	Received Other Systemic Treatment before TKI	1.491	(0.815, 2.727)	0.195
ECOG Score				ECOG Score			
2–3 (vs. 0–1)	4.39	(2.037, 9.458)	0.0002	(2–3 vs. 0–1)	3.435	(1.779, 6.634)	0.0002
Highest Toxicity Grade				Highest Toxicity Grade			
3–5 (vs. 0–2)	1.475	(0.748, 2.909)	0.262	3–5 (vs. 0–2)	0.837	(0.500, 1.400)	0.497
CNS Metastases	0.526	(0.235, 1.177)	0.118	CNS Metastases	0.784	(0.452, 1.358)	0.385
Baseline Mutation				Baseline Mutation			
Exon 19	0.213	(0.048, 0.948)	0.042	Exon 19	0.339	(0.090, 1.309)	0.117
L858R	1.44	(0.331, 6.270)	0.627	L858R	0.732	(0.186, 2.889)	0.656
T790M	0.337	(0.101, 1.125)	0.077	T790M	0.714	(0.280, 1.822)	0.481
G719X	6.239	(1.026, 37.931)	0.047	G719X	1.014	(0.201, 5.110	0.987

1. HR, Hazard Ratio.

## Data Availability

The data that support the findings of this study are available from the corresponding author, M.J.F., upon reasonable request. Data are not made publicly available due to privacy or ethical restrictions.

## Supplementary Materials

The following are available online at https://www.mdpi.com/2072-6694/13/6/1426/s1, Figure S1. Progression free survival probability for NLR < 3 (red) compared to NLR ≥ 3 (blue) at (a) treatment start, (b) 6 weeks of therapy and (c) 12 weeks of therapy, Figure S2: Progression free survival probability for BMI ≤ 18.5 (red), >18.5 to ≤25 (green), >25 to ≤30 (blue) and >30 (purple) at (a) treatment start, (b) 6 weeks of therapy and (c) 12 weeks of therapy. Overall survival probability for BMI ≤ 18.5, >18.5 to ≤25, >25 to ≤30 and >30 at (d) treatment start, (e) 6 weeks of therapy and (f) 12 weeks of therapy, Figure S3: Correlation between change in BMI and change in NLR between treatment initiation and 12 weeks of therapy, Table S1. Progression free survival (PFS) and overall survival (OS) for patients with NLR ≥ 3 and <3 at therapy start, 6 weeks and 12 weeks after beginning treatment, Table S2: Progression free survival (PFS) and overall survival (OS) for patients with BMI ≤ 18.5, >18.5 to ≤25, >25 to ≤30 and >30 at treatment start, 6 weeks after therapy and 12 weeks after therapy and OS at progression for patients with BMI ≤ 18.5, >18.5 to ≤25, >25 to ≤30 and >30, Table S3: Change in BMI on OS and PFS.
