# Supplementary material for: Neutrophil-to-Lymphocyte Ratio Is a Predictive Biomarker in Patients with Epidermal Growth Factor Receptor (EGFR) Mutated Advanced Non-Small Cell Lung Cancer (NSCLC) Treated with Tyrosine Kinase Inhibitor (TKI) Therapy
Source: Cancers (Basel). 2021 Mar 20;13(6):1426. doi: 10.3390/cancers13061426 (PMC8003851; doi:10.3390/cancers13061426)
Supplement: Supplementary file 1 [file cancers-13-01426-s001.pdf]

Supplementary Materials: Neutrophil-to-Lymphocyte Ratio is a Predictive Biomarker in Patients with Epidermal Growth Factor Receptor (EGFR) Mutated Advanced Non-small Cell Lung Cancer (NSCLC) Treated with Tyrosine Kinase Inhibitor (TKI) Therapy

Nicole K. Yun, Sherin J. Rouhani, Christine M. Bestvina, Ethan M. Ritz, Brendan A. Gilmore, Imad Tarhoni, Jeffrey A. Borgia, Marta Batus, Philip D. Bonomi and Mary Jo Fidler

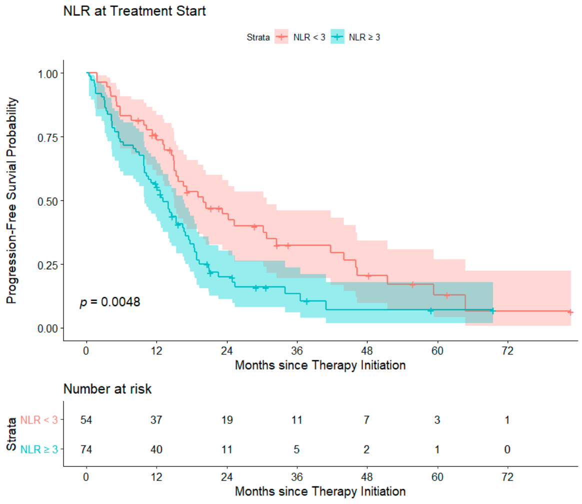

(**a**)

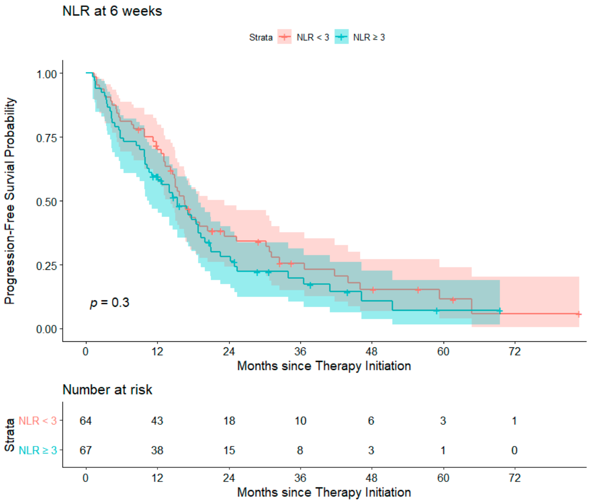

(**b**)

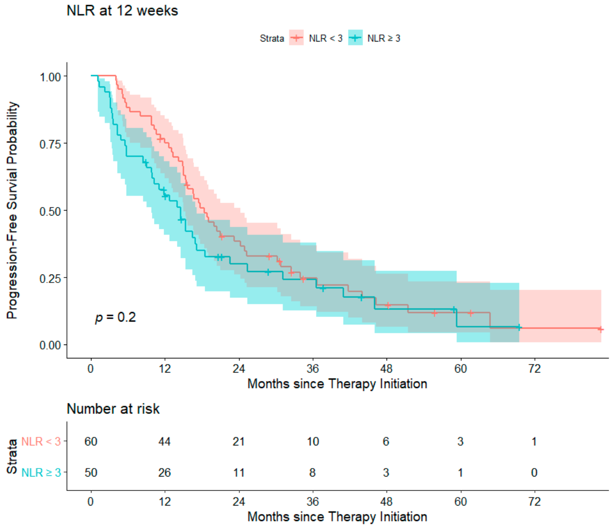

(**c**)

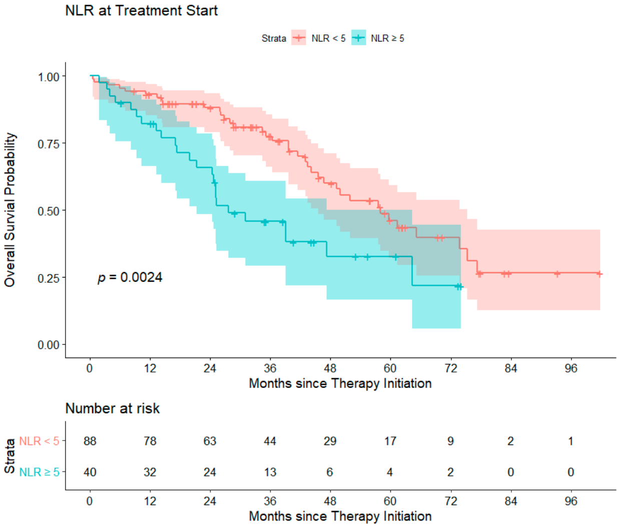

(**d**)

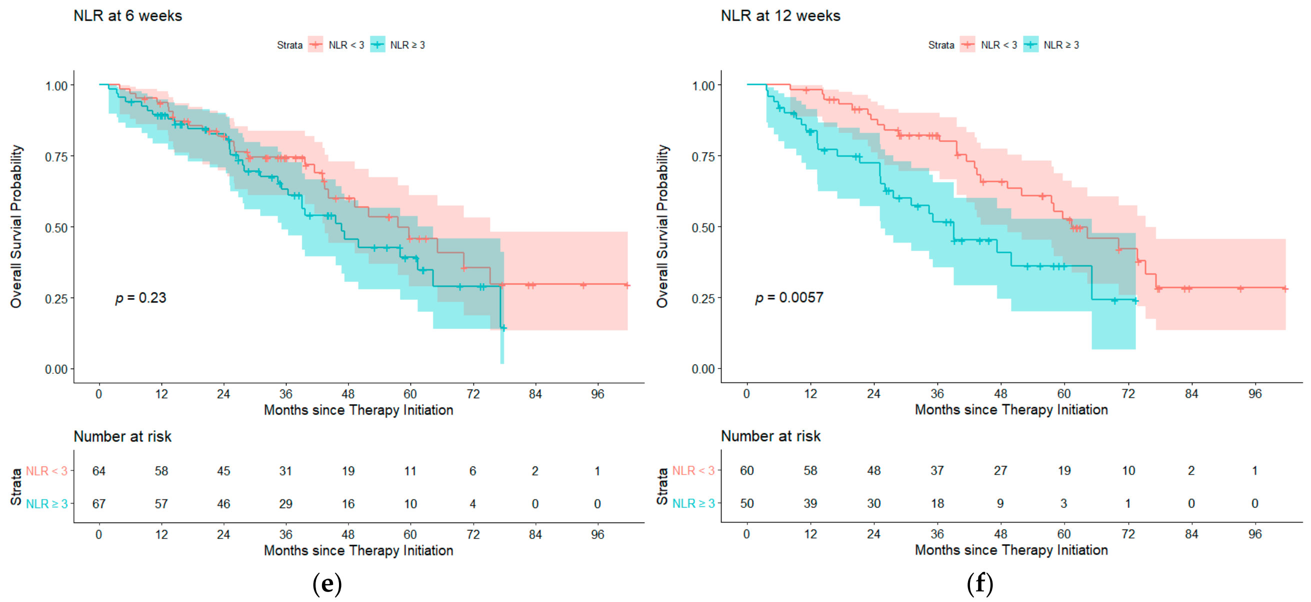

Figure S1. Progression free survival probability for NLR < 3 (red) compared to NLR ≥ 3 (blue) at (**a**) treatment start, (**b**) 6 weeks of therapy and (**c**) 12 weeks of therapy. Overall survival probability for NLR < 3 compared to NLR ≥ 3 at (**d**) treatment start, (**e**) 6 weeks of therapy and (**f**) 12 weeks of therapy.

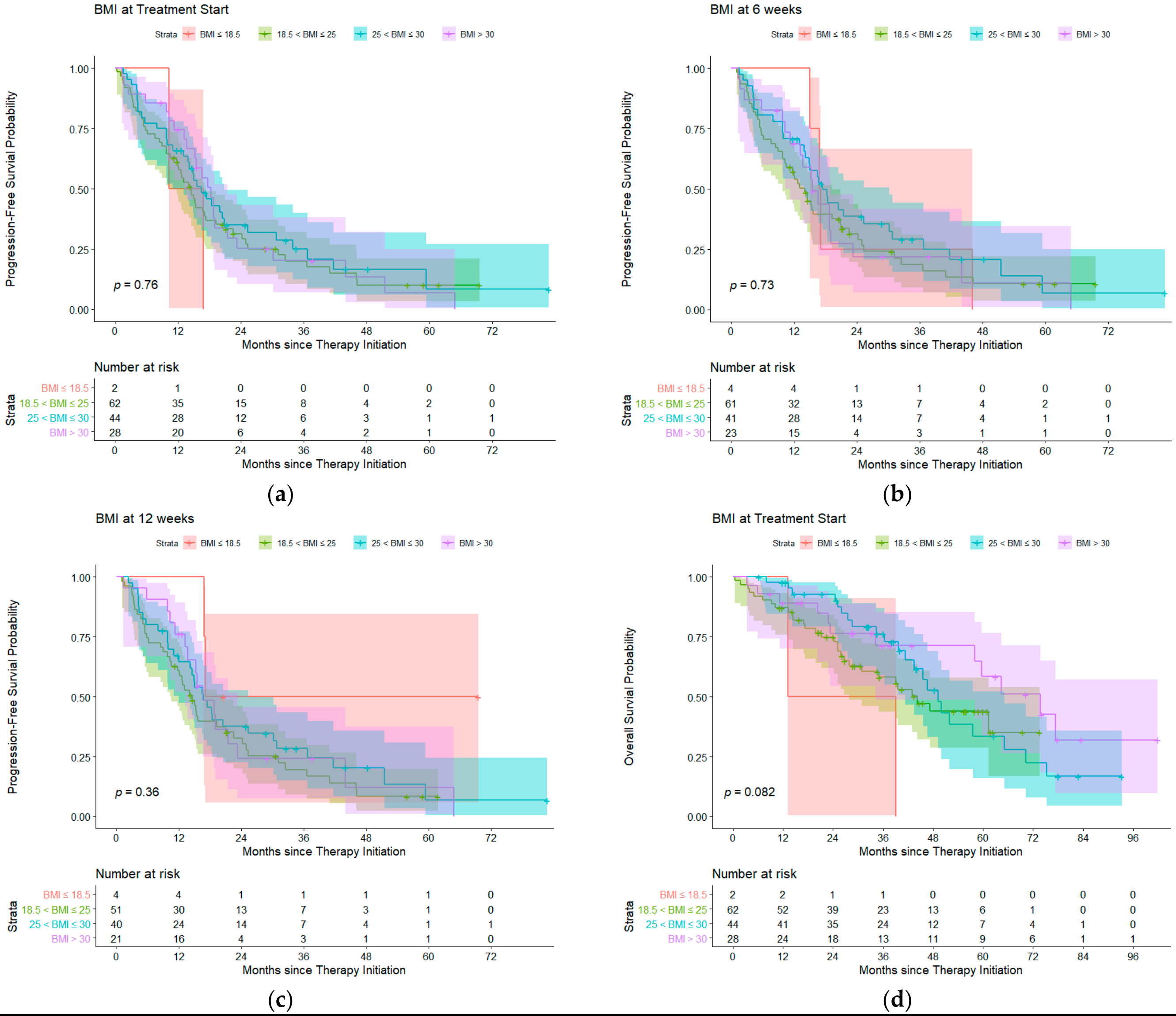

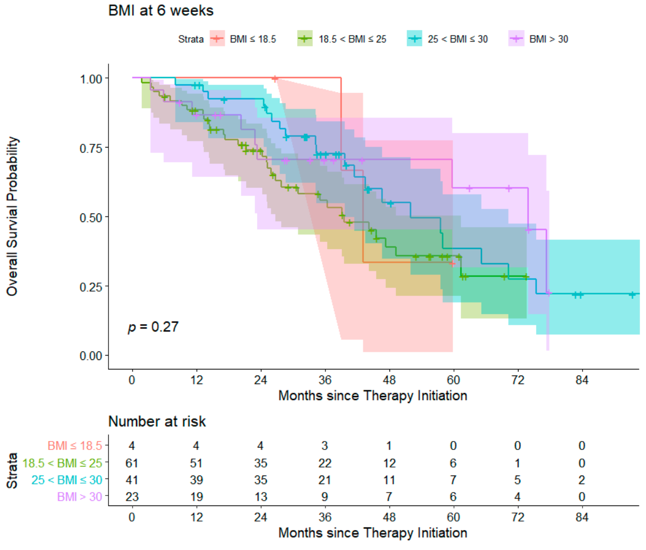

(e)

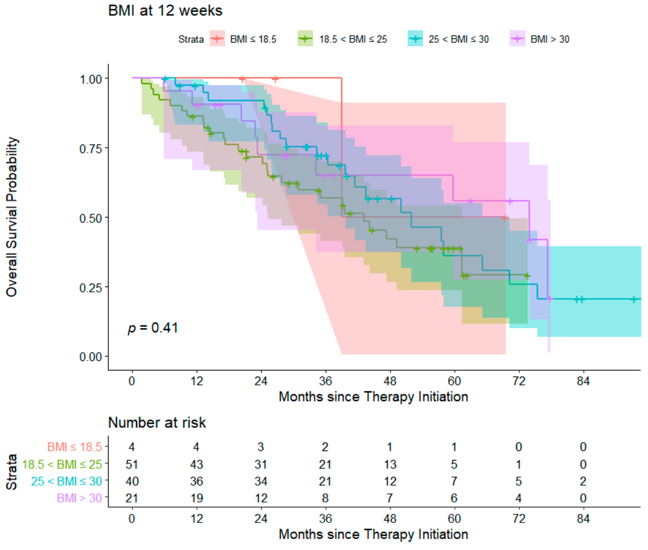

(f)

Figure S2. Progression free survival probability for BMI ≤ 18.5 (red), > 18.5 to ≤ 25 (green), > 25 to ≤ 30 (blue) and > 30 (purple) at **(a)** treatment start, **(b)** 6 weeks of therapy and **(c)** 12 weeks of therapy. Overall survival probability for BMI ≤ 18.5, > 18.5 to ≤ 25, > 25 to ≤ 30 and > 30 at **(d)** treatment start, **(e)** 6 weeks of therapy and **(f)** 12 weeks of therapy.

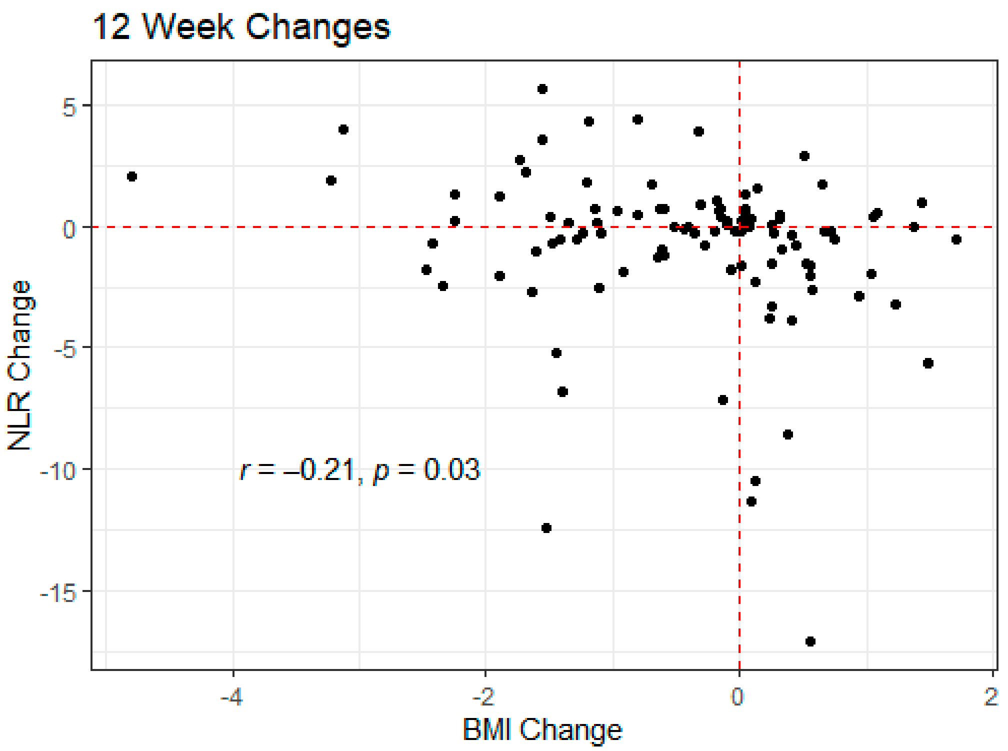

Figure S3. Correlation between change in BMI and change in NLR between treatment initiation and 12 weeks of therapy.

Table S1. Progression free survival (PFS) and overall survival (OS) for patients with NLR ≥ 3 and < 3 at therapy start, 6 weeks and 12 weeks after beginning treatment.

Outcome	NLR	Measurement Time	*n*	Median	95% CI	*p*-value
OS	< 3	Treatment Start	54	61.4	(49.2, 75.3)	0.0029
OS	≥ 3	Treatment Start	74	39.1	(26.7, 47.3)	
OS	< 3	6 weeks after Treatment Start	64	57.6	(43.5, 75.3)	0.23
OS	≥ 3	6 weeks after Treatment Start	67	46.6	(35.1, 64.4)	
OS	< 3	12 weeks after Treatment Start	60	61.4	(49.2, 75.3)	0.0057
OS	≥ 3	12 weeks after Treatment Start	50	39.1	(25.2, 65.1)	
PFS	< 3	Treatment Start	54	20	(15.0, 30.8)	0.0048
PFS	≥ 3	Treatment Start	74	13.1	(9.9, 16.7)	
PFS	< 3	6 weeks after Treatment Start	64	16.6	(14.3, 23.3)	0.3
PFS	≥ 3	6 weeks after Treatment Start	67	15.3	(10.3, 18.8)	
PFS	< 3	12 weeks after Treatment Start	60	18.4	(15.0, 24.3)	0.2
PFS	≥ 3	12 weeks after Treatment Start	50	14.5	(9.8, 17.2)	

Table S2. Progression free survival (PFS) and overall survival (OS) for patients with BMI ≤ 18.5, > 18.5 to ≤ 25, > 25 to ≤ 30 and > 30 at treatment start, 6 weeks after therapy and 12 weeks after therapy and OS at progression for patients with BMI ≤ 18.5, > 18.5 to ≤ 25, > 25 to ≤ 30 and > 30.

Outcome	BMI	Measurement Time	N	Median	95% CI	*p*-value
OS	≤ 18.5	Treatment start	2	26.1	(13.2, N/A)	0.082
OS	> 18.5 to ≤ 25	Treatment start	62	44.2	(27.9, N/A)	
OS	> 25 to ≤ 30	Treatment start	44	49.2	(41.4, 65.1)	
OS	> 30	Treatment start	28	73.8	(34.2, N/A)	
OS	≤ 18.5	6 weeks after treatment start	4	43.0	(39.1, N/A)	0.27
OS	> 18.5 to ≤ 25	6 weeks after treatment start	61	39.6	(26.7, 61.4)	
OS	> 25 to ≤ 30	6 weeks after treatment start	41	51.9	(39.6, 70.2)	
OS	> 30	6 weeks after treatment start	23	73.8	(23.3, N/A)	
OS	≤ 18.5	12 weeks after treatment start	4	39.1	(39.1, N/A)	0.41
OS	> 18.5 to ≤ 25	12 weeks after treatment start	51	43.0	(25.2, 61.4)	
OS	> 25 to ≤ 30	12 weeks after treatment start	40	51.9	(39.6, 70.2)	
OS	> 30	12 weeks after treatment start	21	73.8	(23.3, N/A)	
PFS	≤ 18.5	Treatment start	2	13.6	(10.3, N/A)	0.76
PFS	> 18.5 to ≤ 25	Treatment start	62	14.5	(10.5, 17.4)	
PFS	> 25 to ≤ 30	Treatment start	44	16.5	(11.1, 20.9)	
PFS	> 30	Treatment start	28	18.4	(13.3, 21.5)	
PFS	≤ 18.5	6 weeks after treatment start	4	17.0	(15.0, N/A)	0.73
PFS	> 18.5 to ≤ 25	6 weeks after treatment start	61	14.0	(9.9, 19.3)	
PFS	> 25 to ≤ 30	6 weeks after treatment start	41	17.5	(14.0, 30.2)	
PFS	> 30	6 weeks after treatment start	23	15.5	(11.3, 19.0)	
PFS	≤ 18.5	12 weeks after treatment start	4	17.2	(16.9, N/A)	0.36
PFS	> 18.5 to ≤ 25	12 weeks after treatment start	51	14.5	(10.3, 21.0)	
PFS	> 25 to ≤ 30	12 weeks after treatment start	40	16.7	(12.0, 25.3)	
PFS	> 30	12 weeks after treatment start	21	16.6	(13.3, 23.3)	
OS	≤ 18.5	Progression	7	22.2	(1.6, N/A)	0.057
OS	> 18.5 to ≤ 25	Progression	49	17.9	(12.5, 31.9)	
OS	> 25 to ≤ 30	Progression	33	33.4	(18.9, 38.9)	
OS	> 30	Progression	15	63.5	(4.6, N/A)	

Table S3. Change in BMI on OS and PFS.

Outcome	Predictor	Hazard Ratio	*p*-value
OS	BMI 6-week change	0.7813	0.107
OS	BMI 12-week change	0.8328	0.174
PFS	BMI 6-week change	0.8949	0.331
PFS	BMI 12-week change	0.9252	0.439
OS after progression	BMI change at progression	0.83711	0.00336

Copyright: © 2021 by the authors. Licensee MDPI, Basel, Switzerland. This article is an open access article distributed under the terms and conditions of the Creative Commons Attribution (CC BY) license (http://creativecommons.org/licenses/by/4.0/).
